# Denosumab for dialysis patients with osteoporosis: A cohort study

**DOI:** 10.1038/s41598-020-59143-8

**Published:** 2020-02-12

**Authors:** Kyohei Kunizawa, Rikako Hiramatsu, Junichi Hoshino, Hiroki Mizuno, Yuko Ozawa, Akinari Sekine, Masahiro Kawada, Keiichi Sumida, Eiko Hasegawa, Masayuki Yamanouchi, Noriko Hayami, Tatsuya Suwabe, Naoki Sawa, Yoshifumi Ubara, Kenmei Takaichi

**Affiliations:** 1 Nephrology Center, Toranomon Hospital Kajigaya, Kanagawa, Japan; 2 Department of Nephrology, Kyorin University, Tokyo, Japan; 3 Nephrology Center, Toranomon Hospital, Tokyo, Japan; 4 The Okinaka Memorial Institute for Medical Research, Tokyo, Japan

**Keywords:** Osteoporosis, End-stage renal disease

## Abstract

Evidence for the efficacy of denosumab in HD patients is limited. Accordingly, here we report a study on the safety and efficacy of denosumab in these patients. We prospectively followed 324 patients (121 HD and 203 non-HD patients) receiving denosumab between June 2013 and May 2018, assessing changes in bone mineral density (BMD) and bone metabolic markers, and noting side-effects. Annual changes in BMD at the lumbar spine in HD and non-HD patients from baseline were, respectively, 6.7 ± 11.1% and 7.5 ± 10.2% (*p* = 0.60), those at the femoral neck were 4.3 ± 7.9% and 3.1 ± 9.5% (*p* = 0.32), and those at the distal radius were −0.5 ± 6.4% and 0.2 ± 13.0% (*p* = 0.66). The prevalence of hypocalcemia (<8.5 mg/dL) was significantly higher in HD than in non-HD patients (35.6% vs 5.4%, *p* < 0.001). The median elapsed time between the first injection of denosumab and the occurrence of hypocalcemia was 7 days in HD patients. The decrease of serum calcium was greater in patients with higher TRACP5b, corticosteroid use, and those without CaCO_3_ supplementation. Our study suggests that denosumab was equally as effective in HD as non-HD patients. However, careful hypocalcemia monitoring, for at least 4 weeks, is recommended for HD patients.

## Introduction

Bone fracture is a major complication in patients on hemodialysis (HD). Indeed, it has been reported that the incidence of bone fracture among HD patients is 5–6 times higher than in the general population^1^. Because the efficacy of bisphosphonate treatment has not been extensively investigated in patients with severe renal impairment^2^, treatment options for patients with end-stage renal disease are very limited due to the lack of data regarding efficacy, the risks of drug accumulation, and side-effects^3^.

Recently, superior efficacy of denosumab, a human monoclonal antibody to the receptor activator of nuclear factor kappa-B ligand (RANKL), for the treatment of osteoporosis in the general population has been reported. This followed many studies suggesting that denosumab could increase bone mineral density (BMD) and decrease the risk of bone fractures in both men and women with osteoporosis who are at high risk of fracture^4–6^.

Because it has been reported that renal function impairment has no significant effect on the pharmacokinetic profile of denosumab^2^, the benefit of denosumab in HD patients is expected to be similar to that seen in patients with normal renal function^7,8^. In fact, we previously reported that an increase of BMD was observed in 13 HD patients with osteoporosis^9^. However, a higher incidence of hypocalcemia in HD patients has also been noted^7,8^. Considering the unmet needs of dialysis patients for new osteoporosis treatments, and the pharmacodynamics of denosumab in these patients, clarification of the safety and efficacy of denosumab, and identification of risk factors for hypocalcemia after initiating treatment with this drug in dialysis patients is warranted. Accordingly, we launched a prospective cohort study to analyze the efficacy and safety of the drug, and to identify the factors associated with hypocalcemia after drug initiation.

## Results

Of the 383 patients with osteoporosis who received denosumab in our Department from June 11, 2013 to May 21, 2018, 59 with a history of peritoneal dialysis, renal transplantation, or severe hypercalcemia (≧15.0 mg/dL) were excluded. The remaining patients who could be followed for at least 4 weeks were enrolled in this study as the “calcium cohort” (n = 121 HD and 203 non-HD). The BMD of a subgroup composed of 83 HD and 137 non-HD patients could be analyzed before and one year after treatment (Supplementary Fig. 1). Patient characteristics are shown in Table 1. The mean estimated glomerular filtration rate (eGFR) in the non-HD population was 61 mL/min/1.73 m^2^. For patients with chronic kidney disease (CKD) grades G1 or 2, G3a, G3b, and G4 or 5 (n = 104, 44, 35, and 20, respectively), the mean and interquartile range (IQR) of the dialysis vintage in the HD patients was 13 (5, 22) years. The HD group was younger than the others, with a lower proportion of females, and higher serum phosphate, alkaline phosphatase (ALP), bone alkaline phosphatase (BAP), total procollagen type 1 amino-terminal propeptide (tP1NP), intact parathyroid hormone (iPTH), and titrate-resistant acid phosphatase 5b (TRACP-5b), suggesting higher bone turnover in these patients. However, the baseline BMD at the lumbar spine (LS), femoral neck (FN), and 1/3 distal radius (DR) was similar in both HD and non-HD patients. The dosing schedule for vitamin D and calcium carbonate at the first denosumab injection is shown in Supplementary Table 1. Indications for corticosteroid use were based on patient background disease, which included nephritis, systematic lupus erythematosus, rheumatoid arthritis, vasculitis, sarcoidosis, and bronchial asthma. Cinacalcet, active vitamin D, and bisphosphonate were used for treatment of hyperparathyroidism and/or osteoporosis.

**Table 1:** Patients’ characteristics.

	HD (n = 121)	Non-HD (n = 203)	p-value
Age (years)	66.7 ± 10.6	71.2 ± 10.9	<0.001
Female sex (%)	60.3%	85.0%	<0.001
Height (cm)	157.1 ± 9.3	151.6 ± 8.6	<0.001
Weight (kg)	49.2 ± 11.0	46.9 ± 8.4	0.03
Body mass index (kg/m2)	19.8 ± 3.2	20.4 ± 3.0	0.11
eGFR (mL/min/1.73 m2)		61 [43–74]	
CKD G1:2:3a:3b:4:5 (%)	11:39:22:17:8:2		
Dialysis vintage (years)	13 [5–22]	—	
Corrected Calcium	9.9 ± 0.8	9.6 ± 0.4	<0.01
Serum Phosphate	4.6 ± 1.2	3.5 ± 0.5	<0.001
Alkaline phosphatase (IU/L)	323 ± 218	241 ± 92	<0.001
BAP (μg/L)	19.6 ± 12.4	14.4 ± 6.4	<0.001
total P1NP (μg/L)	141 [73–283]	41 [24–65]	<0.001
Intact PTH (pg/mL)	132 [56–217]	54 [39–74]	<0.001
TRACP-5b (mU/dL)	496 [299–797]	354 [254–481]	<0.001
**Bone Mineral Density (DEXA)**			
Lumbar Spine (T score)	−2.12 ± 1.59	−2.09 ± 1.69	0.91
(BMD (g/cm2))	0.79 ± 0.19	0.76 ± 0.33	0.41
Femoral Neck (T score)	−2.45 ± 1.00	−2.36 ± 1.17	0.46
(BMD (g/cm2))	0.54 ± 0.12	0.54 ± 0.13	0.90
Distal Radius (T score)	−2.81 ± 2.24	−2.54 ± 2.27	0.40
(BMD (g/cm2))	0.53 ± 0.13	0.52 ± 0.16	0.73

Abbreviations: HD, dialysis; BMD, bone mineral density; eGFR, estimated glomerular filtration rate; BAP, bone alkaline phosphatase; P1NP, procollagen type 1 amino-terminal propeptide; iPTH, intact parathyroid hormone; TRACP-5b, titrate-resistant acid phosphatase 5b.

### Changes of BMD after denosumab treatment

**Figure 1:**
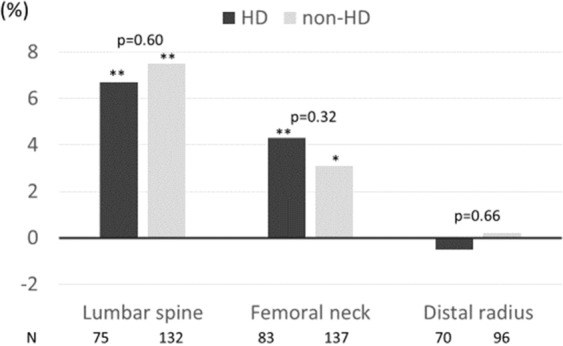
Annual change in bone mineral density in hemodialysis (HD) and non-HD patients. p-values represent comparisons between HD and non-HD groups. p-values of in-group comparison from baseline are shown as *(p < 0.05) or **(p < 0.01).

Figure 1 shows the mean changes in BMD at the LS, FN, and DR in the two groups before and one year after denosumab initiation. BMD at the LS was significantly increased from baseline in both HD (6.7 ± 11.1%, *p* < 0.01) and non-HD patients (7.5 ± 10.2%, *p* < 0.01). In addition, changes of BMD at the FN in HD and non-HD patients from baseline were, respectively, 4.3 ± 7.9% and 3.1 ± 9.5%, and those at the DR were -0.5 ± 6.4% and 0.2 ± 13.0%. These changes of BMD were generally similar in all patients, although the increase in HD patients was slightly smaller at the LS and DR, but slightly greater at the FN, than in the other patients. Data on annual changes of BMD at the LS, FN and DR before denosumab treatment initiation were available for 45 HD patients. Their characteristics were similar to other HD patients except for higher proportions of active vitamin D and bisphosphonate users. Annual changes of BMD at the LS, FN and DR from baseline before and after denosumab were −0.1 ± 7.4% and 5.1 ± 10.5% (*p* = 0.03), −2.4 ± 7.4% and 2.5 ± 7.0% (*p* = 0.03), and −1.7 ± 3.5% and 0.3 ± 4.8% (*p* = 0.08), respectively (Supplementary Tables 2 and 3).

### Changes of bone metabolic markers after denosumab

**Figure 2:**
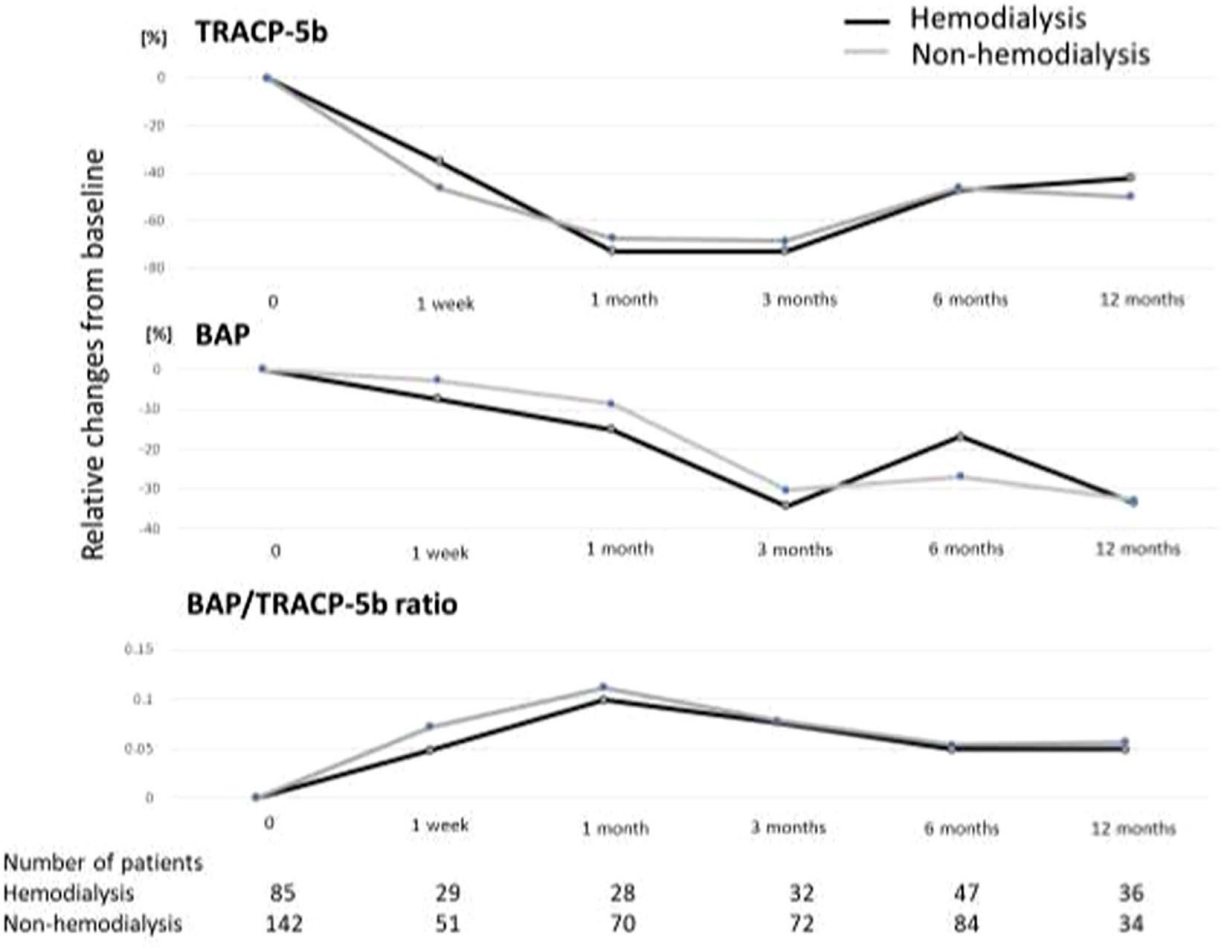
Changes of bone metabolic markers after denosumab in hemodialysis (HD) and non-HD patients. BAP, bone alkaline phosphatase; TRACP5b, titrate-resistant acid phosphatase 5b.

Next, we compared changes of TRACP-5b, BAP, and the ratio of BAP/TRACP5b, in HD and non-HD patients. As shown in Fig. 2, changes of TRACP5b were similar in both groups, with the greatest decrease in values one month after denosumab initiation. Changes in BAP and BAP/TRACP5b ratios were also similar in the two groups, although the BAP/TRACP5b ratio was somewhat lower for HD than for non-HD patients one month after denosumab injection.

### Adverse events: risk of hypocalcemia after denosumab

**Figure 3:**
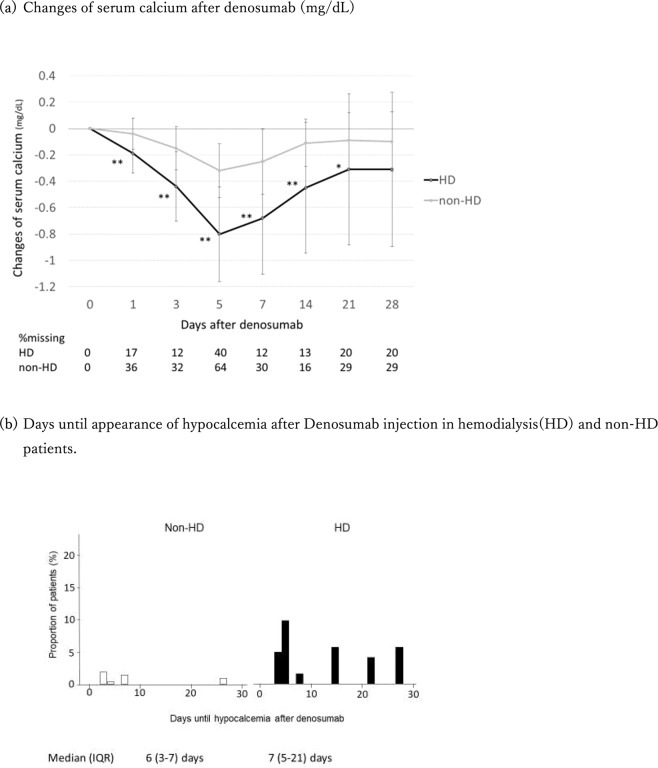
Decline of serum calcium from baseline after denosumab in hemodialysis (HD) and non-HD patients. (**a**) Changes of serum calcium after denosumab (mg/dL). (**b**) Days until appearance of hypocalcemia after denosumab injection in HD and non-HD patients. Hypocalcemia was defined as albumin-corrected serum calcium <8.5 mg/dL. First event of hypocalcemia after denosumab was captured.

None of the patients in our cohort experienced allergy, severe hypophosphatemia, or jaw osteonecrosis, but some experienced hypocalcemia. Declines of albumin-corrected serum calcium (Ca) from baseline in HD and non-HD patients are shown in Fig. 3a. The mean decline in Ca from baseline in HD patients was significantly less than in non-HD patients at day 1 (−0.19 ± 0.30 vs −0.04 ± 0.24 mg/dL, *p* < 0.001), and the lowest at day 5 in both HD and non-HD patients (−0.80 ± 0.72 vs −0.32 ± 0.41 mg/dL, *p* < 0.001).

The incidence of hypocalcemia—defined as Ca < 8.5 mg/dL—was significantly greater in HD than in non-HD patients (35.6% vs 5.4%, *p* < 0.001) (Table 2). Moreover, 22 of 121 (18.2%) HD patients met the criteria for grade 2 hypocalcemia (7.0 ≦ calcium < 8.0 mg/dL), and in nine of them a serum calcium level below 7.5 mg/dL was recorded. One patient with CKD G5 who did not take a calcium/magnesium/cholecalciferol combination tablet met the criteria for grade 3 hypocalcemia, but none of the patients experienced hypocalcemia-related symptoms. The duration of hypocalcemia after drug injection in HD patients varied from 3 to 28 days with a mean of 7 days and IQR of 5–21 days (Fig. 3b).

**Table 2:** Patients and grades of hypocalcemia after denosumab use.

Grade* of the lowest serum calcium	Non-HD (n = 203)				HD	p-value
	CKD G1,2 (n = 104)	CKDG3a (n = 44)	CKDG3b (n = 35)	CKDG4,5 (n = 20)	(n = 121)	
Grade 1 (8.0 ≦ Ca < 8.5)	1(1.0%)	1(2.3%)	3(8.6%)	5 (25%)	21(17%)	0.05
Grade 2 (7.0 ≦ Ca < 8.0)	0	0	0	0	22(18%)	0.003
Grade 3–5 (Ca < 7.0)	0	0	0	1(5%)**	0	n/a

HD, dialysis; n/a, not assessed.

All calcium was adjusted by serum albumin.

*Common Terminology Criteria for Adverse Events (CTCAE) version 4.0.

**One patient who did not take calcium/magnesium/cholecalciferol combination tablet before injection.

**Figure 4:**
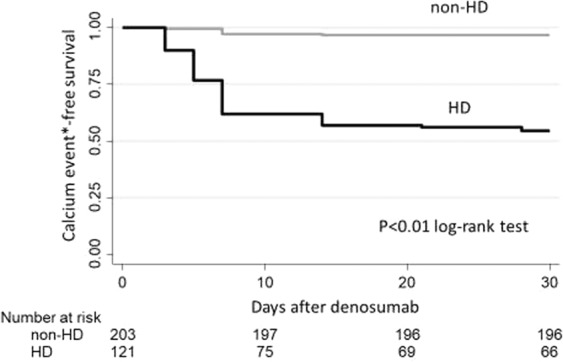
Comparison of Kaplan-Meier curves for incidence of hypocalcemia* in HD and non-HD patients. *Hypocalcemia was defined as decline of calcium >1.5 mg/dL or >1.0 mg/dL after an increased dose of active vitamin D and/or calcium carbonate.

The proportion of patients who experienced large decreases in calcium levels, defined as a decline of calcium >1.5 mg/dL or >1.0 mg/dL after an increased dose of active vitamin D and/or calcium carbonate after denosumab injection, was significantly higher among HD than non-HD patients (45.5% vs 3.5%, *p* < 0.01). In most cases, this decline occurred within 10 days after injection (Fig. 4). These results suggest that for HD patients, careful management after drug use is needed, in particular for the first 10 days and continuing for at least a month thereafter, even if their active vitamin D dose is increased (or newly started). The proportions of patients administered increased doses of vitamin D and/or calcium carbonate are shown in Supplementary Table 4.

### Factors associated with calcium decline after denosumab treatment of HD patients

We analyzed factors associated with calcium decline in HD patients. Table 3 shows the IQR of maximum calcium decline divided by quartiles of age, dialysis vintage, ALP, BAP, tP1NP, iPTH, and TRACP5b levels, and categorized by sex and use of active vitamin D, calcium carbonate (CaCO_3_), bisphosphonates, cinacalcet, and corticosteroid. These data suggest that patients with a shorter dialysis vintage, higher BAP, tP1NP, and TRACP5b levels, and patients without CaCO_3_ supplementation or on corticosteroid treatment, experienced a larger decrease in calcium after denosumab injection (median, ≧1.5 mg/dL).

**Table 3:** Comparison of the maximum calcium decline from baseline after denosumab injection in dialysis patients.

Variables	Quartile 1	Quartile 2	Quartile 3	Quartile 4	p-value
Age	0.60 (1.30)	0.85 (1.40)	1.15 (1.35)	1.30 (1.10)	0.78
Dialysis vintage	1.55 (1.30)*	0.85 (1.40)	0.60 (1.30)	0.60 (1.25)	**0.02**
ALP	0.65 (1.20)	0.55 (1.35)	1.50 (1.30)	1.40 (0.70)	0.13
BAP	0.40 (1.30)	0.70 (1.10)	1.35 (1.30)	1.60 (0.50)*	**0.005**
tP1NP	0.20 (1.06)	1.10 (1.40)**	1.10 (0.95)**	1.50 (0.80)**	**0.006**
iPTH	1.20 (1.80)	0.65 (1.10)	0.60 (1.40)	1.40 (1.30)	0.08
TRACP5b	0.50 (1.10)	1.10 (1.00)	0.70 (1.30)	1.50 (0.50)**	**0.02**
	Yes		No		
Female	1.10 (1.50)		0.80 (1.20)		0.34
Active vitamin D	1.10 (1.20)		0.60 (1.30)		0.41
Calcium carbonate	0.70 (1.20)		1.50 (1.25)*		0.08
Bisphosphonate	0.40 (1.30)		0.95 (1.30)		0.45
Cinacalcet	0.80 (1.00)		1.20 (1.40)		0.57
Corticosteroid	**1.50 (1.40)**		0.85 (1.30)		0.53

Median (Interquartile range).

Quartile 1 is the smallest value group.

*p < 0.05, **p < 0.01, p-values comparing to the lowest group:

Abbreviations:

HD, dialysis; ALP, alkaline phosphatase; BAP, bone alkaline phosphatase; tP1NP, total procollagen type 1 amino-terminal propeptide; iPTH, intact parathyroid hormone; TRACP5b, titrate-resistant acid phosphatase 5b.

Serum calcium was adjusted by serum albumin.

Next, we estimated the hazard ratios (HRs) for marked calcium decline, defined as a maximum calcium decline ≧1.5 mg/dL before an increased dose of active vitamin D and/or CaCO_3_ was given, or ≧1.0 mg/dL even after an increased dose, for each variable. Because the correlation coefficients between 3 bone formation markers (ALP, BAP, and tP1NP) were large, we compared the log-likelihoods by univariate analyses, and chose tP1NP as the most suitable of the three for inclusion in multivariate analyses. After adjusting for age, sex, body mass index, dialysis vintage, tP1NP, iPTH, use of active vitamin D, CaCO_3_, bisphosphonates, cinacalcet, and corticosteroid, we found that the highest quartile of TRACP5b (sex-specific) was associated with a large decrease of calcium in model 1 when using the lowest quartile as the reference (HR 3.93 [1.51, 10.24]) (Table 4). Other variables associated with a large decrease in calcium were use of CaCO_3_ with an HR of 0.48 (0.25, 0.94), and use of corticosteroid with an HR of 3.09 (1.10, 8.66). Similar findings were obtained in model 2. Thus, patients with higher TRACP5b, corticosteroid use, and those without CaCO_3_ use, were considered at high risk for marked calcium declines after denosumab treatment.

**Table 4:** Factors associated with large decreases of calcium* after denosumab in HD patients.

Variables		Univariate	p	Multivariate (Model 1)	p	Multivariate (Model 2)	p
		HR (95% CI)		HR (95% CI)		HR (95% CI)	
Age (years)		1.01 (0.98, 1.04)	0.44			1.01 (0.97, 1.06)	0.53
Sex (female)		1.00 (0.58, 1.72)	0.99	0.46 (0.22, 0.95)	0.04	0.45 (0.18, 1.15)	0.10
Body mass index (kg/m2)		0.96 (0.88, 1.05)	0.38	0.94 (0.85, 1.04)	0.25	0.92 (0.81, 1.06)	0.24
Dialysis vintage (≦5years)	Q1	1.00		1.00		1.00	
(5.1–15 years)	Q2	0.96 (0.47, 1.92)	0.90	1.77 (0.74, 4.24)	0.20	2.77 (0.88, 8.74)	0.08
(15.1–22 years)	Q3	0.75 (0.38, 1.49)	0.41	0.76 (0.32, 1.83)	0.54	0.66 (0.20, 2.21)	0.50
(>22 years)	Q4	0.45 (0.19, 1.03)	0.06	0.53 (0.20, 1.44)	0.22	0.68 (0.17, 2.71)	0.58
tP1NP(μg/L) (≦62.52)	Q1	1.00				1.00	
(62.53–119.06)	Q2	1.06 (0.38, 2.93)	0.91			0.66 (0.15, 2.99)	0.59
(119.07–246.00)	Q3	1.53 (0.58, 4.04)	0.39			1.15 (0.27, 4.91)	0.86
(>246.00)	Q4	1.86 (0.73,4.73)	0.19			1.77 (0.41, 7.76)	0.45
Intact PTH (logarithmic)		1.17 (0.84, 1.63)	0.35			0.78 (0.50, 1.21)	0.27
TRACP5b (mU/dL) (M, ≦ 305; F ≦ 290)	Q1	1.00		1.00		1.00	
(M, 391.1–565; F, 290.1–487)	Q2	1.62 (0.60, 4.36)	0.34	2.36 (0.83, 6.73)	0.11	3.56 (1.01, 12.51)	0.048
(M, 565.1–904; F, 487.1–751)	Q3	1.46 (0.54, 3.92)	0.45	1.62 (0.59, 4.47)	0.35	2.24 (0.60, 8.42)	0.23
(M, >904; F, >751)	Q4	2.86 (1.16, 7.05)	0.02	3.93 (1.51, 10.24))	<0.01	3.86 (1.01, 14.83)	0.049
Active vitamin D		0.91 (0.36, 2.28)	0.84			0.91 (0.14, 5.79)	0.92
Calcium carbonate		0.50 (0.30, 0.86)	0.01	0.48 (0.25, 0.94)	0.03	0.49 (0.20, 1.20)	0.12
Bisphosphonate		0.68 (0.17, 2.81)	0.60			0.49 (0.20, 2.27)	0.34
Cinacalcet		0.97 (0.55, 1.68)	0.90			1.88 (0.65, 5.42)	0.25
Corticosteroid		2.07 (0.93, 4.57)	0.07	3.09 (1.10, 8.66)	0.03	5.22 (1.04, 26.30)	0.04

Abbreviations: HR, hazard ratio; CI, confidence interval; p, p-value; tP1NP, total procollagen type 1 amino-terminal propeptide; iPTH, intact parathyroid hormone; TRACP5b, titrate-resistant acid phosphatase 5b (sex specific: M, male; F, female).

Serum calcium was adjusted by serum albumin.

Intact PTH was log-transformed.

Model 1, backward stepwise. Other variables were removed due to p > 0.20. model 2, full adjustment.

## Discussion

In this study, we found that denosumab increased BMD of the LS and FN in HD patients, and that these effects were similar to those in non-HD patients. Nakamura *et al*. reported that in patients with normal renal function, annual changes of BMD were 6.6% at the LS, 2.8% at the FN, and 0.2% at the DR^10^. These results are very similar to ours in the non-HD population. For HD patients, a recent meta-analysis reported that there were significant increases in T-scores with mean differences of 0.39 (0.10, 0.69) for the LS and 0.79 (0.60, 0.98) for the FN and significant reductions in ALP and iPTH 4–12 months after denosumab treatment^8^. In that paper, improvement of BMD was higher in FN than LS. However, 2 of 3 papers reporting changes of BMD in meta-analyses included enrolled patients with severe hyperparathyroidism. Thus, differences in patient characteristics may have caused the discrepancy for changes at the FN. In fact, it was also reported that improvement of BMD in FN was relatively greater in patients with severe hyperparathyroidism (iPTH > 800 pg/mL) than in those with moderate hyperparathyroidism^11^. We also found that changes in TRACP5b and BAP after denosumab injection were similar in HD and non-HD patients. The BAP/TRACP5b ratio increased continuously up to 1 month after denosumab injection, and then decreased, although the ratio at 1 month was slightly lower in HD than in non-HD patients. Block *et al*. reported that mean serum denosumab concentrations over time after subcutaneous administration of 60 mg denosumab were similar in patients with different degrees of renal dysfunction, including dialysis patients^2^. These findings imply that the pharmacodynamics and pharmacokinetics of denosumab in HD and non-HD patients are similar.

In terms of prevention of bone fracture, it has been reported that relative to placebo denosumab reduces the relative risk of new fractures by 68% for vertebrae, 40% for hips, and 20% for other non-vertebral sites in postmenopausal women^5^. Moreover, these preventive effects persisted for 3–8 years after treatment^4,12^. A similar preventive effect was observed among the CKD G1-4 sub-groups^7^. However, it is not clear at this point whether improvement of BMD actually does translate into preventing bone fractures in HD patients, because low BMD is only a small part of the spectrum of renal osteodystrophy in such patients. Moreover, the safety and efficacy of inhibition of osteoclast activity over the long term is also not clear in HD patients. Therefore, longer follow-up studies to assess the preventive effects on bone fracture, or bone biopsy studies, are needed to confirm whether denosumab is indeed useful in this respect.

In terms of safety, we did find that the risk of hypocalcemia due to denosumab was significantly higher in HD patients than in those not on dialysis, with the median time of occurrence being at 7 days. Moreover, it is notable that severe hypocalcemia was seen even in patients who were prescribed active vitamin D and/or CaCO_3_. In fact, the study by Block *et al*. reported that 5 of 8 (63%) HD patients and 3 of 9 (33%) patients with severe CKD experienced hypocalcemia of <8.0 mg/dL2. It follows that for denosumab use in HD patients, frequent monitoring of serum calcium, for example three times a week, is necessary at least for the first week, and preferably for a month, with dose increases or addition of active vitamin D and CaCO_3_ supplementation as appropriate. In addition, our study found that the occurrence of hypocalcemia and decrease in serum calcium were associated with higher baseline TRACP5b levels, even after adjusting for possible confounders. We therefore posit that baseline TRACP5b could be a predictor of denosumab-induced hypocalcemia in HD patients. Although there is a broad spectrum of disordered bone turnover in HD patients, it is generally considered that turnover is higher than in the general population. Consistent with this, the median TRACP5b level in HD patients was significantly higher than in non-HD patients in our study. In addition, corticosteroid is known to decrease bone formation and increase bone resorption^13^. Given the increase in bone resorption in these patients, it is reasonable to assume that the risk of hypocalcemia after denosumab treatment was higher among this group because the endogenous supply of calcium from bone is rapidly reduced by the drug, whereas deposition of mineral into new matrix remains increased^2^.

There are several limitations to this study. First, because of the intrinsic nature of observational studies, we were unable to confirm the efficacy and safety of denosumab in HD patients. In addition, the data in this study are incomplete, which may weaken our conclusion. For example, the frequency of measurement of serum calcium levels up to day 28 was significantly higher in HD patients than in non-HD patients, which may have biased the recording of episodes of hypocalcemia. However, limiting our analysis to those patients with serum calcium measures recorded at least five times over 28 days resulted in data almost identical to previous reports (data not shown). Another limitation of the present study is that the observed improvement in BMD may not be an effect of denosumab alone, but could be caused by the combined effects of denosumab, vitamin D (active or native), and calcium use. Other clinical trials involving HD patients are needed to confirm causation. In addition, there may be selection bias in the present study, despite the fact that indication bias for denosumab use is probably minimal because the main variable was independent of each patient’s intention of receiving dialysis. However, there may still have ben some selection bias because our primary analyses were performed only on those patients whose BMD data were both available before and at one year after denosumab use. Another limitation is that our results can be extrapolated only to those patients with adequate iPTH control, and might not apply to other patients, such as those with severe hyperparathyroidism or hypoparathyroidism, including a history of parathyroidectomy. Although the effect of denosumab and calcitriol on severe secondary hyperparathyroidism in HD patients has been reported^11^, adequate control of iPTH by phosphate binders, active vitamin D, and/or cinacalcet should be considered before denosumab use, as recommended in the guidelines^14,15^. In fact, a recent study suggested that improvement of bone histomorphometry was still observed after treatment with cinacalcet in HD patients who had secondary hyperparathyroidism^16^. Finally, more severe aortic calcification in HD patients and other factors, such as physical activity or serum magnesium levels, may affect any increase of BMD. To minimize the effect of aortic calcification on measurements of lumbar BMD, we measured front and side lumbar BMD in all patients. The improvement in the BMD of HD patients was found to be similar to that of non-HD patients despite the expected lower physical activity of most HD patients. Therefore, potential differences between the two groups in terms of physical activity and all the other factors mentioned above are not likely to change the conclusions from this study.

Thus, our study showed that denosumab is effective for the treatment of osteoporosis in HD patients, increasing BMD to a degree similar to that seen in patients not on dialysis. Careful monitoring of serum calcium is necessary in HD patients due to the high risk of hypocalcemia, and baseline serum TRACP5b could be a potential predictor of hypocalcemia in these patients after denosumab injection. Considering the unmet needs of HD patients for osteoporosis treatment, and its superior efficacy, it is reasonable to assume that denosumab may become a first- or second-line choice for management of these patients. Our results contribute to reducing the likelihood of bone fracture in this high-risk population and to improving their quality of life.

## Methods

### Study participants

This was a prospective, observational, open-label study launched at two centers starting in June 2013. The study protocol and the waiver of informed consent were approved by the Ethics Committee of Toranomon Hospital (Approval No. 737). The study was conducted in accordance with the Declaration of Helsinki and the ethical guidelines for epidemiological research in Japan. All adult CKD patients with or without dialysis in our Department who had osteoporosis and who began denosumab treatment between June 2013 and May 2018 were enrolled in this study. Thus, we included all CKD patients and did not select by any other criteria. The definition of osteoporosis was based on criteria published by the Japanese Osteoporosis Society^15^. Briefly, patients were diagnosed with osteoporosis if their BMD was <70% of the young adult mean (YAM) or their T-score was <−2.5 at the LS at L2-4, the FN, and the DR as measured by dual-energy X-ray absorptiometry (DEXA) (Hologic, Waltham, MA), or if they were categorized as being at high risk of bone fracture with BMD < 80% of the YAM or a T-score < −1.0 in the LS and FN. Criteria for assigning high risk were a previous history of fragility fracture, a family history of bone fracture in the proximal femur, or having a risk of bone fracture during the past 10 years of >15% according to the Japanese fracture risk assessment tool^17^.

### Study design and procedures

Patients were screened for baseline evaluations prior to denosumab injection. They were excluded if they had a history of malignancy or parathyroidectomy, had active infection, were hypocalcemic (Ca < 8.5 mg/dL), or were pregnant at screening. It was recommended that patients with hyperparathyroidism (iPTH > 110 pg/mL in CKD G3-5 and iPTH > 240 pg/mL in CKDG5) be given medications for this condition before denosumab injection in accordance with previous reports and standard guidelines^2,18,19^.

Eligible patients received a subcutaneous dose of 60 mg denosumab (Pralia in Japan and Prolia in the US and EU; Amgen, Inc., Thousand Oaks, CA, USA). It was recommended that all patients, especially those on HD, stay in hospital for 5–10 days to monitor serum Ca levels and any other adverse effects, and that they return for follow-up visits every month. Patients who were followed for at least one month were analyzed for changes in their serum markers; those who were followed for a year with their BMD assessed were analyzed for changes in BMD. At each visit, any adverse effects were recorded and fasting blood samples were obtained. History of medication use was taken, including doses of oral/intravenous active vitamin D, CaCO_3_, non-Ca-containing phosphate binders and cinacalcet. For patients not on dialysis, the recommendation was that they should start combination tablets (Denotas, Daiichi-Sankyo Co., Tokyo, Japan) that included 1,525 mg of calcium carbonate (610 mg calcium), 400 IU of cholecalciferol, and 118.4 mg of magnesium carbonate (30 mg magnesium) on the same day as the denosumab injection instead of their previous active vitamin D or calcium drugs. HD patients were recommended to start or increase their doses of active vitamin D (usually 0.25 to 1.0 μg/day of oral alfacalcidol) before or on the same day as the denosumab injection. During the study period, administration of active vitamin D and/or calcium was based on clinical judgement. None of the HD patients received ergocalciferol or cholecalciferol during this study. Denosumab was injected subcutaneously every 6 months. All HD patients followed a dialysis schedule of 4-hour sessions three times a week with a dialysate calcium concentration of 2.5 mEq/L.

### Laboratory measurements

All patients were scheduled for DEXA measurement of BMD (LS, FN, and DR) before and one year after treatment. Lateral lumbar BMD was assessed instead of posteroanterior lumbar BMD if patients had severe aortic calcifications. Serum Ca, phosphate, creatinine, ALP and use of drugs including active vitamin D and calcium carbonate, were scheduled for measurement at days 0, 1, 3, 5, 7, 14, 21, and 28. If serum albumin was lower than 4.0 mg/dL, serum Ca was adjusted by serum albumin using Payne’s equation (adjusted calcium = calcium -albumin + 4.0). BAP, iPTH, TRACP-5b, and tP1NP were quantified at day 0 and 7, and then at 1, 3, 6, and 12 months. Intact P1NP values were converted to tP1NP values based on the equation tP1NP = 1.03 × iP1NP − 1.45, according to the manufacturer’s protocol (patients with osteoporosis, n = 50, r = 0.98)^20^. Serum iPTH and tP1NP levels were measured by electrochemiluminescence immunoassay (Roche Diagnostics K.K., Tokyo, Japan). BAP was measured by chemiluminescence immunoassay (Beckman Coulter Inc., Indianapolis, IN, USA). Intact-P1NP was measured by radioimmunoassay (Orion Diagnostica, Espoo, Finland), and TRACP-5b was measured by enzyme immunoassay (DS Pharma Biomedical, Osaka, Japan).

### Definition of outcomes

The primary purpose of our registry was to evaluate changes of BMD, while the second purpose was to clarify the safety of denosumab in CKD patients. The severity of hypocalcemia was defined by the Common Terminology Criteria for Adverse Events, version 4.0, as follows: grade 1, 8.0 ≦ Ca < 8.5 mg/dL; grade 2, 7.0 ≦ Ca < 8.0 mg/dL; grade 3, 6.0 ≦ Ca < 7.0 mg/dL; grade 4, Ca < 6.0 mg/dL; and grade 5, death^21^. We also evaluated the maximum calcium decline, which was calculated as the difference between baseline calcium level and the minimum serum calcium level at any time throughout the observation period. Because one of our aims was to identify factors associated with calcium decline from baseline and to determine baseline calcium level variations in HD patients, we defined a large decline in serum calcium as the maximum calcium decline from the baseline ≧1.0 mg/dL even after an increased dose of active vitamin D or CaCO_3_, or ≧1.5 mg/dL, regardless of any change in these medications.

### Statistical analyses

Patient characteristics are reported for patients with or without dialysis. Normally-distributed variables are summarized as the means ± SDs, and non-normally distributed variables as medians and IQRs. Intact PTH levels were log-transformed to normalize the distribution. For comparison of unpaired data, the Chi-squared or Fisher’s exact test was used for categorical variables, the unpaired t-test or Mann-Whitney U test was used for continuous variables. The paired t-test or Wilcoxon signed rank test was used for paired continuous variables. For multiple comparisons, analysis of variance was used for parametric variables, and the Kruskal-Wallis test was used for non-parametric variables. The regression substitution approach was used to compensate for missing data during the follow-up period.

Maximum calcium decline was compared according to the subgroups of each baseline factor. Continuous variables such as age, dialysis vintage, etc. were categorized using quartiles for the subgroup comparison. Hazard ratios for large declines in serum calcium were calculated using the Cox proportional hazard model. The backward stepwise method was used for the selection of variables in the multivariate model (model 1), and all possible confounders were adjusted in model 2. The proportional hazard assumption was tested by log-log plot and Schoenfeld residuals. All analyses were performed with STATA SE software, version 14.2 (StataCorp LLC, College Station, TX, USA).

## Supplementary information

Supplementary tables and figure.
