# Supplementary material for: Denosumab for dialysis patients with osteoporosis: A cohort study
Source: Sci Rep. 2020 Feb 12;10:2496. doi: 10.1038/s41598-020-59143-8 (PMC7016112; doi:10.1038/s41598-020-59143-8)
Supplement: Supplementary file 1 — Supplementary tables and figure. [file 41598_2020_59143_MOESM1_ESM.pdf]

Denosumab for dialysis patients with osteoporosis: A cohort study

Kyohei Kunizawa[a,b], Rikako Hiramatsu[a], Junichi Hoshino*[a,c,d], Hiroki Mizuno[c], Yuko Ozawa[c], Akinari Sekine[c], Masahiro Kawada[c], Keiichi Sumida[a], Eiko Hasegawa[c], Masayuki Yamanouchi[a], Noriko Hayami[a], Tatsuya Suwabe[a], Naoki Sawa[a], Yoshifumi Ubara[a,d], and Kenmei Takaichi[a,c,d]

a Nephrology Center, Toranomon Hospital Kajigaya, Kanagawa, Japan
b Department of Nephrology, Kyorin University, Tokyo, Japan
c Nephrology Center, Toranomon Hospital, Tokyo, Japan
d The Okinaka Memorial Institute for Medical Research, Tokyo, Japan

Supplementary table 1

Distribution of doses of vitamin D and calcium carbonate at the first injection of denosumab

(a) Dialysis patients (n=121)

Vitamin D	Daily doses of calcium carbonate				Total
	Zero	0.5-<1.5g	1.5-<3.0g	≧3.0g	
Active, oral	30%	8%	10%	22%	70%
Active, injection	9%	3%	4%	5%	22%
No vitamin D	4%	1%	0%	3%	8%
Total	44%	11%	14%	31%	100%

(b) Non dialysis patients (n=203)

Vitamin D	Daily doses of calcium carbonate				Total
	Zero	0.5-<1.5g	1.5-<3.0g or Ca/VD/Mg tablets	≧3.0g	
Natural, oral	0%	0%	35%	0%	35%
Active, oral	50%	0%	6%	0%	56%
No vitamin D	9%	0.5%	0%	0%	9%
Total	59.5%	0.5%	41%	0%	100%

Abbreviation: HD, hemodialysis; NA: not analyzed because of no patient in the cell.

*Ca/VD/Mg tablets: combination of 1,525mg of calcium carbonate, 400 IU of cholecalciferol, and 118.4mg of magnesium

Supplementary table 2

(a) Changes of bone mineral density (BMD) before and after denosumab treatment

Year after Denosumab	N	Before<br>1 year	After<br>1 year	p-value
Lumbar spine (BMD%)<br>(T-score)	45	-0.1±7.4%<br>(-0.04±0.48)	5.1±10.5%<br>(0.35±0.62)	0.03
Femoral neck (BMD%)<br>(T-score)	45	-2.4±7.4%<br>(-0.14±0.35)	2.5±7.0%<br>(0.10±0.30)	0.03
Distal radius (BMD%)<br>(T-score)	37	-1.7±3.5%<br>(-0.19±0.38)	0.3±4.8%<br>(0.43±2.31)	0.08

(b) Changes of laboratory values before and after denosumab treatment

Time after denosumab	N	Before<br>1 year	Before<br>6 months	Denosumab<br>injection	After<br>6 months	After<br>1 year
Alkaline phosphatase (IU/L)	45	283±111	294±106	311±141	247±117	252±133
Intact parathyroid hormone (pg/mL)	43	168[75-304]	111[24-172]	124[55-212]	107[38-198]	51[26-97]

Supplementary table 3

Comparison of patients on dialysis with and without bone mineral density measurement 1 year before denosumab injection.

	With (n=45)	Without (n=76)	p-value
**Age (y)**	68.1±9.5	65.8±11.1	0.25
**Female sex (%)**	64.4%	57.9%	0.48
**Body mass index (kg/m^2)**	19.9±2.7	19.5±4.2	0.52
**Dialysis vintage (years)**	13.7±11.3	15.9±11.8	0.32
**Corrected Calcium**	9.6±0.6	10.0±0.8	<0.01
**Serum Phosphate**	4.4±1.2	4.8±1.2	0.16
**Alkaline phosphatase (IU/L)**	311±329	329±251	0.66
**BAP (μg/L)**	20.3±12.2	19.2±12.7	0.67
**total P1NP (μg/L)**	118 [60-290]	150 [85-271]	0.68
**Intact PTH (pg/mL)**	124 [55-212]	135 [63-241]	0.74
**TRACP-5b (mU/dL)**	423 [297-716]	547 [369-821]	0.28
Bone Mineral Density (DEXA)			
**Lumbar Spine (T score)**	-1.86±1.66	-2.29±1.51	0.19
**(BMD (g/cm^2))**	0.82±0.21	0.76±0.18	0.14
**Femoral Neck (T score)**	-2.57±0.89	-2.38±1.08	0.33
**(BMD (g/cm^2))**	0.52±0.11	0.55±0.14	0.29
**Distal Radius (T score)**	-2.66±2.33	-2.86±2.15	0.69
**(BMD (g/cm^2))**	0.53±0.12	0.53±0.13	0.81
Active vitamin D	97.7%	87.8%	0.06
Calcium carbonate	61.4%	51.4%	0.30
Bisphosphonate	11.1%	1.4%	0.02
Cinacalcet	40.9%	31.0%	0.28
Corticosteroid	8.9%	7.9%	0.85

Abbreviations: HD, dialysis; BMD, bone mineral density; eGFR, estimated glomerular filtration ratio; BAP, bone alkaline phosphatase; P1NP, procollagen type 1 amino-terminal propeptide; iPTH, intact parathyroid hormone; TRACP-5b, titrate-resistant acid phosphatase 5b

Supplementary table 4

Proportions of patients who increased dose of vitamin D and calcium carbonate

(a) Dialysis patients (n=121)

Vitamin D	Daily doses of calcium carbonate				Total
	Zero	0.5-<1.5g	1.5-<3.0g	≧3.0g	
Active, oral	84% *(20%)*	11% *(11%)*	45% *(27%)*	54% *(8%)*	49% *(16%)*
Active, injection	73% *(18%)*	67% *(33%)*	80% *(20%)*	33% *(0%)*	64% *(16%)*
No vitamin D	20% *(0%)*	0% *(100%)*	NA	75% *(0%)*	40% *(10%)*
Total	57% *(18%)*	23% *(23%)*	56% *(25%)*	53% *(6%)*	52% *(16%)*

(b) Non dialysis patients (n=203)

Vitamin D	Daily doses of calcium carbonate				Total
	Zero	0.5-<1.5g	1.5-<3.0g or Ca/VD/Mg tablets	≧3.0g	
Natural, oral	NA	NA	6% *(0%)*	NA	6% *(0%)*
Active, oral	19% *(2%)*	NA	17% *(8%)*	NA	19% *(3%)*
No vitamin D	22% *(6%)*	0% *(0%)*	NA	NA	21% *(5%)*
Total	19% *(3%)*	0% *(0%)*	7% *(1%)*	NA	14% *(2%)*

The percentage represents proportion of patients who increased dose of vitamin D and those who increased dose of CaCO3 (in parentheses, italic) in each cell.

Abbreviation: HD, hemodialysis; NA: not analyzed because of no patient in the cell.

*Ca/VD/Mg tablets: combination of 1,525mg of calcium carbonate, 400 IU of cholecalciferol, and 118.4mg of magnesium

Supplementary figure 1

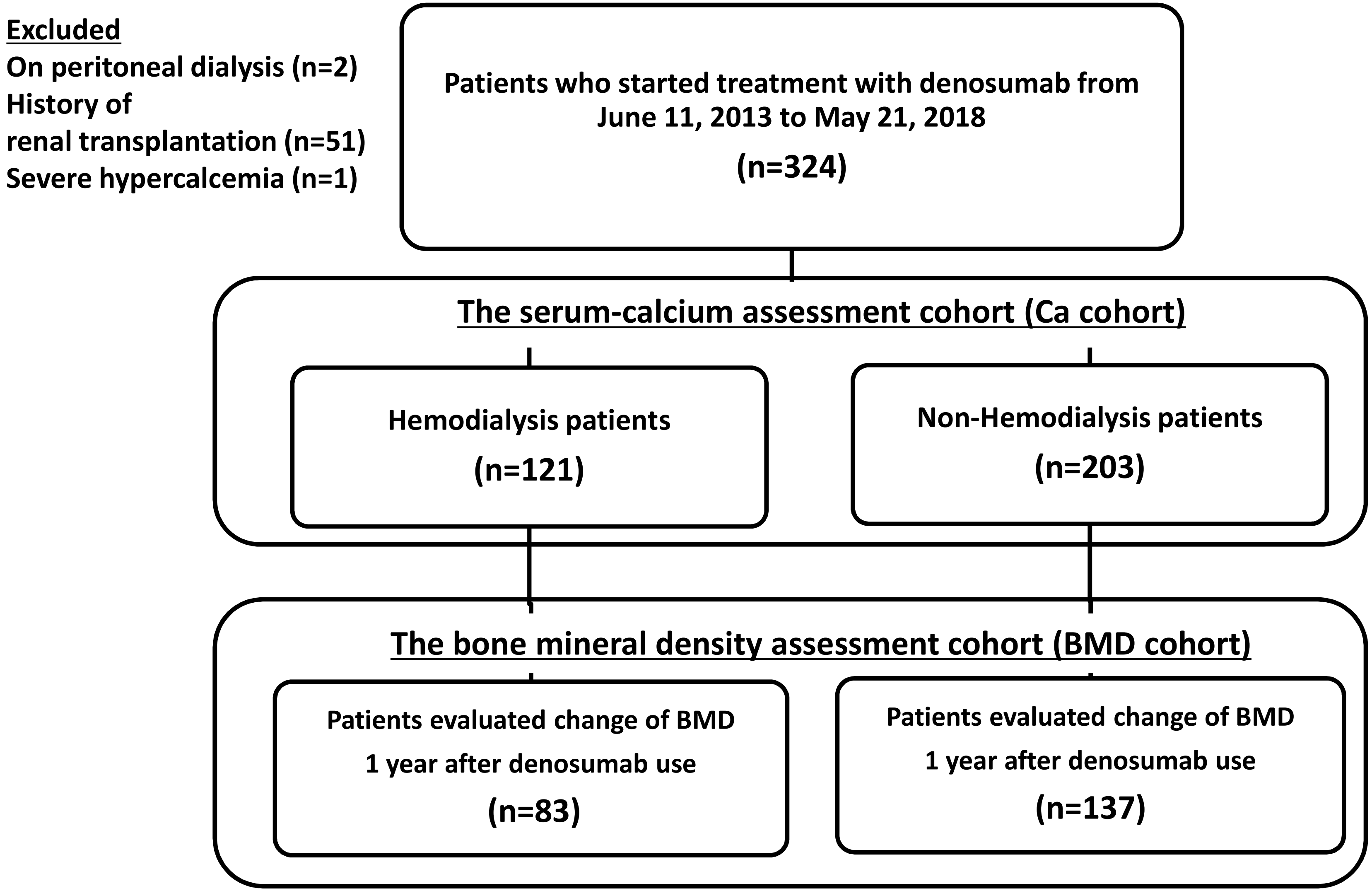

BMD, bone mineral density
